# NADPH Oxidase 4: Crucial for Endothelial Function under Hypoxia—Complementing Prostacyclin

**DOI:** 10.3390/antiox13101178

**Published:** 2024-09-27

**Authors:** Heike Brendel, Jennifer Mittag, Anja Hofmann, Helene Hempel, Sindy Giebe, Patrick Diaba-Nuhoho, Steffen Wolk, Christian Reeps, Henning Morawietz, Coy Brunssen

**Affiliations:** 1 Division of Vascular Endothelium and Microcirculation, Department of Medicine III, Faculty of Medicine and University Hospital Carl Gustav Carus, TUD Dresden University of Technology, 01307 Dresden, Germany; jennifer.mittag@ukdd.de (J.M.); h.hempel@gross-sand.de (H.H.); sindy.giebe@uniklinikum-dresden.de (S.G.); patrick.diabanuhoho@ukmuenster.de (P.D.-N.); coy.brunssen@ukdd.de (C.B.); 2 Division of Vascular and Endovascular Surgery, Department of Visceral, Thoracic and Vascular Surgery, Faculty of Medicine and University Hospital Carl Gustav Carus, TUD Dresden University of Technology, 01307 Dresden, Germany; anja.hofmann2@ukdd.de (A.H.); steffen.wolk@uniklinikum-dresden.de (S.W.); christian.reeps@ukdd.de (C.R.); 3 Department of Paediatric and Adolescent Medicine, Paediatric Haematology and Oncology, University Hospital Münster, 48149 Münster, Germany

**Keywords:** NADPH oxidase 4, hypoxia, PTGIS, endothelial function, human endothelial cells, laminar flow

## Abstract

*Aim*: The primary endothelial NADPH oxidase isoform 4 (NOX4) is notably induced during hypoxia, with emerging evidence suggesting its vasoprotective role through H_2_O_2_ production. Therefore, we aimed to elucidate NOX4′s significance in endothelial function under hypoxia. *Methods*: Human vessels, in addition to murine vessels from *Nox4^−/−^* mice, were explored. On a functional level, Mulvany myograph experiments were performed. To obtain mechanistical insights, human endothelial cells were cultured under hypoxia with inhibitors of hypoxia-inducible factors. Additionally, endothelial cells were cultured under combined hypoxia and laminar shear stress conditions. *Results*: In human occluded vessels, NOX4 expression strongly correlated with prostaglandin I2 synthase (*PTGIS*). Hypoxia significantly elevated NOX4 and PTGIS expression and activity in human endothelial cells. Inhibition of prolyl hydroxylase domain (PHD) enzymes, which stabilize hypoxia-inducible factors (HIFs), increased NOX4 and PTGIS expression even under normoxic conditions. *NOX4* mRNA expression was reduced by HIF1a inhibition, while *PTGIS* mRNA expression was only affected by the inhibition of HIF2a under hypoxia. Endothelial function assessments revealed hypoxia-induced endothelial dysfunction in mesenteric arteries from wild-type mice. Mesenteric arteries from *Nox4^−/−^* mice exhibited an altered endothelial function under hypoxia, most prominent in the presence of cyclooxygenase inhibitor diclofenac to exclude the impact of prostacyclin. Restored protective laminar shear stress, as it might occur after thrombolysis, angioplasty, or stenting, attenuated the hypoxic response in endothelial cells, reducing HIF1a expression and its target *NOX4* while enhancing *eNOS* expression. *Conclusions*: Hypoxia strongly induces NOX4 and PTGIS, with a close correlation between both factors in occluded, hypoxic human vessels. This relationship ensured endothelium-dependent vasodilation under hypoxic conditions. Protective laminar blood flow restores eNOS expression and mitigates the hypoxic response on NOX4 and PTGIS.

## 1. Introduction

Inadequate oxygen supply is a characteristic aspect of many diseases, in particular those of the cardiovascular system. Hypoxia resulting from a pathophysiological decline in oxygen tension or an interruption in blood flow activates a regulatory network of transcription factors and signaling proteins coordinating adaptations in cellular function, metabolism, proliferation, DNA repair, and apoptosis [1,2].

Most cellular responses to hypoxia are mediated by hypoxia-inducible factors (HIFs). These heterodimeric transcription factors are able to regulate a great number of genes in response to lower oxygen levels or hypoxic stress [3]. Under hypoxic conditions, a functional dimer of the corresponding α and β subunits is formed and translocated into the nucleus. There, it recruits co-activators, binds to specific hypoxia-responsive elements (HREs), and regulates the transcription of HIF target genes. Oxygen-sensing prolyl hydroxylase domain enzymes (PHDs) target HIFa subunits for proteasomal degradation in normoxia through hydroxylation [4]. This hydroxylation is critical for the HIFa subunits to be recognized by the von Hippel–Lindau (VHL) protein which, as part of the pVHL–elongin B-C (VBC) complex, links the subunits with ubiquitin and thus targets them for proteosomal degradation. Under hypoxic conditions, the prolyl hydroxylases responsible for the hydroxylation are inhibited; therefore, the HIFa subunits are not degraded but stabilized in sufficient amount to allow dimerization and transcription of HIF-dependent genes [5].

Several studies support a crosstalk between hypoxia and NADPH oxidases 4 (NOX4). Thus, hypoxia has been shown to induce NOX4 in kidney [6], brain [7], lung, pulmonary artery smooth muscle cells, and adventitial fibroblasts from patients with pulmonary arterial hypertension [8,9]. NOX4 is one of the seven mammalian NADPH oxidase isoforms. NADPH oxidases are key contributors to the production of reactive oxygen species in the vessel wall [10,11] and are implicated in various cardiovascular diseases. For example, they play a role in aging-related development of endothelial dysfunction [12] and contribute to vascular complications in diabetes [13]. NOX4, in particular, is distinct from other NADPH oxidases due to its predominant production of H_2_O_2_ rather than superoxide anion. Additionally, NOX4 is the main isoform in endothelial cells [14].

It has been reported that NOX4 is inducible by prostaglandin I2 (PGI_2_), commonly known as prostacyclin, signaling [15]. PGI_2_, a potent vasodilator and inhibitor of platelet aggregation, plays a crucial role in maintaining vascular homeostasis. It is produced from arachidonic acid through the actions of cyclooxygenases (COX) and prostaglandin I2 synthase (PTGIS) and is primarily released from the endothelium and smooth muscle cells [16]. PGI_2_ exerts its biological effects on target cells classically through prostaglandin I2 receptor (PTGIR), operating via the cyclic adenosine monophosphate (cAMP)/protein kinase A (PKA)/cAMP response element-binding protein (CREB) pathway [17]. Although PGI_2_’s role in vasodilation under hypoxia has been recognized [18], a relationship between NOX4 and PGI_2_ under hypoxic conditions has not been established. Furthermore, it is perplexing why endothelial cells would utilize oxygen to produce hydrogen peroxide under hypoxia. Our study aimed to elucidate the role of NOX4 under hypoxia, particularly in relation to PGI_2_.

## 2. Materials and Methods

### 2.1. Mouse Models

All experiments were conducted in compliance with the US National Institutes of Health’s Guide for the Care and Use of Laboratory Animals (NIH Publication No. 85–23, revised 1996). The animal research ethics committee of the Technische Universität Dresden and Landesdirektion Sachsen approved the experiments in accordance with institutional guidelines and German animal welfare regulations (AZ: 24-9168.11-1/2012-44, AZ: DD24.1-5131/449/20, AZ: DD24.1-5131/450/9).

C57BL/6J/Nox4^−/−^ (Nox4^−/−^) mice were generously provided by Ralf P. Brandes and Katrin Schröder (Goethe University, Frankfurt, Germany) and bred in-house [19]. As control mice, C57Bl/6J/Nox4^flox/flox^ mice of a wild-type (WT) phenotype were used.

### 2.2. Murine Tissue Sampling

Mice were sacrificed by cervical dislocation; aorta and mesenteric arteries were excised, rinsed with Krebs–Henseleit buffer, and immediately further processed for Amplex Red or vascular function analysis. For RNA isolation, vessels were rinsed with ice-cold PBS and snap-frozen for further analyses.

### 2.3. Recruitment of Patients and Preparation of Internal Mammary Artery

Patients scheduled for elective coronary artery bypass grafting surgery at the Heart Center Dresden were enrolled in the study. All participants received comprehensive verbal and written information about the study and provided written informed consent. The study received approval from the Ethics Committee of the Technische Universität Dresden (EK 307-12-2007) and was conducted in accordance with the principles outlined in the Declaration of Helsinki. Details on gender, age, and clinical characteristics of the patients have been published elsewhere [20].

During coronary artery bypass graft surgery, distal remnant specimens of the left internal mammary artery were collected. The tissue samples were immediately stored at 4 °C and then transported to the laboratory. Under the microscope, the internal mammary artery was carefully separated from connective and perivascular tissue and snap-frozen for future analysis.

### 2.4. Patients with Peripheral Arterial Disease (PAD)

Patients with peripheral arterial disease were admitted to the Division of Vascular and Endovascular Surgery at the Department of Visceral, Thoracic and Vascular Surgery, University Hospital Dresden. During surgery, specimens of the artery wall proximal to the occlusion were obtained. The arterial walls were harvested intraoperatively during peripheral bypass surgery. The examined full-wall artery specimen from the femoral artery was taken after clamping off the blood flow and arteriotomy of the anastomosis site of the bypass.

The Ethics Committee of the Technische Universität Dresden (EK 151-04-2017) granted approval for the study. The investigation fulfils the principles outlined in the Declaration of Helsinki. Informed consent was received from each patient. Details on gender, age, and clinical characteristics of patients were obtained, and percentages are listed below in Table 1. The samples were placed in ice-cold DPBS, dissected, and rapidly frozen in liquid nitrogen. Approximately 10–15 min elapsed between surgical removal and tissue processing.

### 2.5. Cell Culture of Primary Human Endothelial Cells

Isolated primary cultures of human umbilical vein endothelial cells (HUVECs) were pooled and cultured on gelatine-coated plates in M199 medium supplemented with 10% (*v*/*v*) fetal calf serum and 1% (*v*/*v*) growth supplement derived from calf retina. The study was conducted in accordance with the principles of the Helsinki Declaration and was approved by the Ethical Review Board of the Medical Faculty of the TU Dresden (EK124082003). Once the cells reached confluence, they were treated. Dimethyloxallyl glycine (DMOG), a cell-permeable competitive inhibitor of prolyl hydroxylase, stabilizes HIFa expression under normal oxygen tension in cultured cells. HUVECs were stimulated with 10 mM DMOG for 24 h.

For hypoxia induction, cells were cultured in a multi-gas O_2_/CO_2_ incubator at 37 °C. Nitrogen gas was introduced into the chambers to create a controlled reduction in the oxygen percentage. Hypoxia experiments were performed at 1% (*v*/*v*) O_2_, 5% (*v*/*v*) CO_2_, and 94% (*v*/*v*) N_2_. For normoxia, cells were cultured in incubators with 5% (*v*/*v*) CO_2_ and 21% (*v*/*v*) O_2_. HUVECs were treated with 300 µM N^G^-nitro-L-arginine methyl ester (L-NAME), a non-selective inhibitor of nitric oxide synthase, for 24 h under normoxic or hypoxic conditions. Cyclooxygenase inhibitor diclofenac was used in a concentration of 10 µM on HUVECs. For HIFa inhibitor experiments, 15 mM HIF1a inhibitor CAS 934593-90-5 (Sigma-Adrich, St. Louis, MO, USA) [21] and 10 µM HIF2a inhibitor CAS 882268-69-1 (Sigma-Adrich, St. Louis, MO, USA) [22] were used.

### 2.6. Lentiviral Downregulation of NOX4

Lentiviral transduction of human endothelial cells was performed as described in [23]. Lentiviral particles containing shNOX4 and shC (scrambled shRNA control) were used for transduction of HUVECs. Transfected cells were selected by medium containing puromycin (1.5 µg/mL) and used for further experiments. The efficiency of NOX4 downregulation was determined via real-time PCR.

### 2.7. mRNA Stability Assay

The mRNA stability was analyzed by treatment with the inhibitor of de novo transcription actinomycin D. HUVECs were exposed to normoxia or hypoxia for 16 h. Next, HUVECs were treated with 5 µg/mL actinomycin D for an additional 8 h under normoxic or hypoxic conditions. After treatment, the cells were immediately lysed, RNA was isolated and reverse transcribed, and NOX4 gene expression was quantified by real-time PCR.

### 2.8. Flow Application on Endothelial Cells

Laminar flow was applied on endothelial cells using either cone-and-plate viscometer [24] or ibidi pump system (ibidi, Martinsried, Germany). For application of high laminar flow, 30 dyn/cm^2^ for 24 h was used.

### 2.9. Griess Assay for Nitrite

Nitrite levels were measured using the Griess reaction. To begin, 100 µL of supernatant from endothelial cells was mixed with 50 µL of 2% (*w*/*v*) aminobenzenesulphoamide in 2.5% (*v*/*v*) phosphoric acid and incubated in the dark for 5 min. Following this, 0.2% (*w*/*v*) NED-reagent (N-(1-naphthyl) ethylenediamine dihydrochloride) was added and the mixture was incubated, protected from light, for 10 min. Absorbance was then measured at 540°nm using a 96-well plate reader. Nitrite concentrations in the samples were calculated using a nitrite standard curve (0–100 µM) and linear regression. Measurements were performed in triplicates.

### 2.10. Detection of Extracellular H_2_O_2_ Using Amplex Red Assay

An Amplex Red Hydrogen Peroxide/Peroxidase Assay Kit was used to measure hydrogen peroxide release (Life Technologies, Darmstadt, Germany). Freshly dissected murine mesenteric arteries of around 3 mm (6 arteries from each mouse) were incubated in Krebs–Henseleit buffer under either normoxic (3 arteries each mouse) or hypoxic conditions (3 arteries). H_2_O_2_-mediated oxidation of Amplex Red and subsequent formation of red fluorescence was measured in a microplate reader (FLUOstar, BMG Labtech, Ortenberg, Germany) as fluorescence (excitation at 544 nm and emission at 590 nm) protected from light.

Regarding H_2_O_2_ measurement from endothelial cells, Amplex Red (50 µM) and horse-radish peroxidase (0.1 U/mL) were added to a colorless M199 cell culture medium. After 30 min, the oxidation of Amplex Red dependent on H_2_O_2_ was measured in the cell culture supernatant. A freshly prepared stock solution of H_2_O_2_ (2 µM) and reaction buffer served as positive and negative controls. The fluorescence was normalized to the protein content of each sample.

### 2.11. Detection of 6-Keto-PGF_1_ Alpha

Prostacyclin (PGI_2_) has a half-life of only 2 to 3 min in buffer. The production of PGI_2_ is therefore commonly monitored by measuring the 6-keto-PGF_1_ alpha concentrations. 6-keto-PGF_1_ alpha is the product of non-enzymatic hydration of PGI_2_ and is more stable. Quantitative measurement of 6-keto-PGF_1_ alpha was performed using an ab133023 ELISA kit (Abcam Limited, Cambridge, UK) and following the manual instructions.

### 2.12. Real-Time PCR

Tissue samples were homogenized using Precellys homogenizer (VWR, peqlab, Erlangen, Germany). Then, total RNA was isolated using peqGOLD (VWR, peqlab, Erlangen, Germany). From treated cells, total RNA was isolated using a High Pure RNA Isolation Kit (Roche Diagnostics, Mannheim, Germany). Reverse transcription was carried out with SuperScript II Reverse Transcriptase, following the manufacturer’s guidelines (Thermo Fisher Scientific, Waltham, MA, USA), using 500 ng of total RNA and random hexamer primers. Quantification was performed by real-time PCR with GoTaq qPCR Master Mix (Promega, Mannheim, Germany). Raw data analysis was conducted with 7500 Software Version 2.06 (Applied Biosystems by Life Technologies, Darmstadt, Germany). Data evaluation was based on a mathematical model of relative expression ratio in real-time PCR with constant reference gene expression [25]. The primers used are listed in Table 2. *POLRIIa*, *TBP*, and *B2m* were used as reference genes.

### 2.13. Western Blot

Proteins were extracted using RIPA Buffer (Cell Signalling, Leiden, The Netherlands) supplemented with Protease Inhibitor Cocktail (Sigma-Aldrich, Munich, Germany). Protein concentration was measured using BCA Protein Assay Reagent (Perbio Science, Bonn, Germany). Proteins (15 µg/lane) were separated by SDS-PAGE and transferred to nitrocellulose membranes (Amersham, Munich, Germany). The membranes were incubated with primary antibodies, as listed in Table 3. The primary antibodies were then detected using secondary horseradish peroxidase-conjugated anti-rabbit or anti-mouse IgG (Zytomed, Berlin, Germany). Protein expression was visualized with Western Lighting Chemiluminescence Reagent Plus (PerkinElmer LAS, Rodgau, Germany) and quantified with ImageJ 1.53t (Java 1.8.0_322) software.

### 2.14. Vascular Function Analysis under Hypoxia

Vascular function analysis was conducted using DMT Multi Wire Myograph System–Model 620 M (Danish Myo Technology, Hinnerup, Denmark). Murine mesenteric arteries were isolated and mounted on wires for isometric tension recordings. The organ bath contained Krebs–Henseleit buffer (4.7 mM KCl, 118 mM NaCl, 1.17 mM MgSO_4_, 1.18 mM KH_2_PO_4_, 25 mM NaHCO_3_, 10 mM glucose, 0.026 mM EDTA, and 1.6 mM CaCl_2_) bubbled with 5% (*v*/*v*) CO_2_ in O_2_. For hypoxic conditions, the organ bath was stirred with 5% (*v*/*v*) CO_2_ in N_2_. Vessels were then equilibrated at 37 °C. Contraction was induced with 80 mM high-potassium Krebs–Henseleit buffer. Following several washes, phenylephrine-induced contraction was assessed. Endothelium-dependent function was assessed by precontracting rings with phenylephrine and relaxing them with increasing concentrations of acetylcholine (0.001–30 µM ACh). To evaluate H_2_O_2_-dependent endothelial function, catalase (1500 U/mL) was added to mesenteric arteries 30 min prior to precontraction. Additionally, 10 µM diclofenac was used on vessel segments to measure impact of cyclooxygenase-catalyzed proteinoids on vascular function. Finally, sodium nitroprusside (30 µM SNP) was added to precontracted vessels for smooth muscle function.

### 2.15. Statistical Analyses

Data are presented as mean ± standard deviation (SD). Normal (Gaussian) distribution was tested by Shapiro–Wilk normality tests. Correlational analysis in non-Gaussian distributed data was conducted using Spearman’s correlation coefficient (r_S_). In normally distributed data, correlation was analyzed using Pearson’s correlation coefficient (r_P_). Non-Gaussian distributed data were analyzed by Kruskal–Wallis and Dunn’s multiple comparison tests. Gaussian distributed data were analyzed with *t*-tests or one-way ANOVAs followed by Tukey’s multiple comparisons test or two-way ANOVA and Sidak’s multiple comparisons tests, respectively (GraphPad Prism 9, GraphPad Software, Inc., La Jolla, CA, USA). A value of *p* < 0.05 was considered as statistically significant.

## 3. Results

### 3.1. NOX4 mRNA Expression Highly Correlated with PTGIS Expression in Occluded Arterial Walls of Patients with Peripheral Arterial Disease

**Figure 1:**
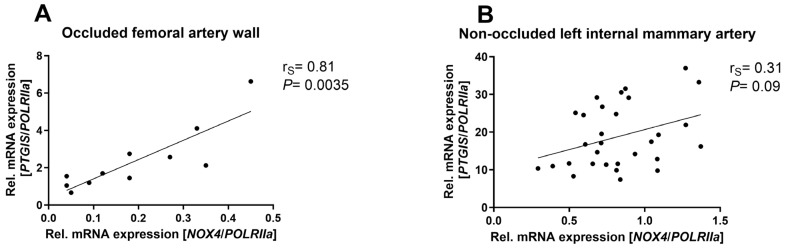
***NOX4* highly correlates with *PTGIS* in human occluded vessels.** (**A**) Correlation of *NOX4* mRNA expression and prostaglandin I2 synthase (*PTGIS*) in femoral artery walls from patients with peripheral arterial disease (*n* = 11). *POLRIIa* mRNA expression was used as reference. (**B**) Correlation of *NOX4* mRNA expression and prostaglandin I2 synthase (*PTGIS*) in human non-occluded left internal mammary arteries (*n* = 30). *POLRIIa* mRNA expression was used as reference. Statistics: Spearman’s correlation coefficient (r_S_) between *PTGIS* and *NOX4* expression. Abbreviations: NOX4, NADPH oxidase 4; PTGIS, prostaglandin I2 synthase; POLRIIa, RNA polymerase II subunit A.

We correlated the expression of *NOX4* mRNA with prostaglandin I2 synthase (*PTGIS*) expression in human vessels. From patients with peripheral arterial disease, specimens of the artery wall proximal to the occlusion were obtained. In those arterial walls, where hypoxia is very likely, we observed a tight correlation between *NOX4* expression and *PTGIS* mRNA expression (Figure 1A). The second specimens analyzed were non-occluded left internal mammary arteries from patients undergoing coronary artery bypass graft surgery. Those arteries were not affected by occlusions. Here, no correlation between *NOX4* and *PTGIS* mRNA expression could be found (Figure 1B).

### 3.2. Hypoxia Elevated NOX4 and PTGIS in Human Endothelial Cells and Murine Vessels

**Figure 2:**
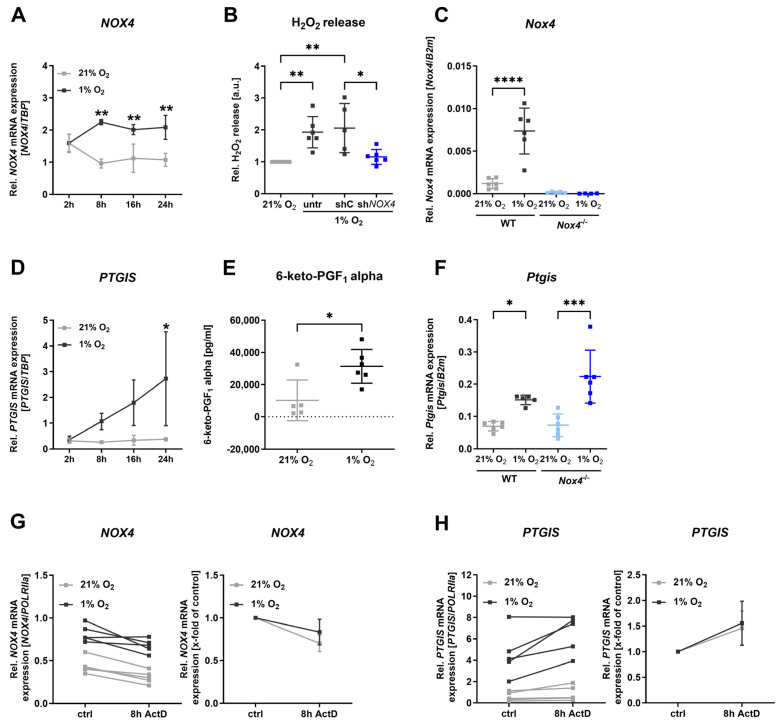
**Increased NOX4 and PTGIS expression after hypoxia in human endothelial cells and murine aorta.** (**A**) *NOX4* mRNA expression after 8 h, 16 h, and 24 h normoxia (21% oxygen) or hypoxia (1% oxygen) (*n* = 3). *TBP* mRNA expression was used as reference. (**B**) Hydrogen peroxide production in HUVECs subjected to transduction with lentiviral particles containing sh*NOX4* and exposed to 24 h hypoxia (1% oxygen) (*n* ≥ 5). Untransduced cells (untr) and cells transduced with scrambled shRNA (shC) were used as controls. (**C**) Relative *Nox4* mRNA expression in aorta from WT and Nox4^−/−^ mice after 24 h hypoxia (1% oxygen) (*n* = 6). *B2m* mRNA expression was used as reference. (**D**) *PTGIS* mRNA expression after 8 h, 16 h, and 24 h normoxia (21% oxygen) or hypoxia (1% oxygen) (*n* = 4). *TBP* mRNA expression was used as reference. (**E**) *6-keto-PGF1 alpha concentration* in supernatant of HUVECs exposed to 24 h hypoxia (1% oxygen) (*n* ≥ 5). (**F**) Relative *Ptgis* mRNA expression in aorta from WT and Nox4^−/−^ mice after 24 h hypoxia (1% oxygen) (*n* = 6). *B2m* mRNA expression was used as reference. (**G**) Relative *NOX4* mRNA expression in HUVECs treated subsequently with actinomycin D (ActD) for 8 h under normoxia and hypoxia (1% oxygen) (*n* = 5). *POLRIIa* mRNA expression was used as reference. Mean relative *NOX4* mRNA expression in HUVECs treated subsequently with actinomycin D (ActD) for 8 h under normoxia and hypoxia (1% oxygen) (*n* = 5). *POLRIIa* mRNA expression was used as reference. (**H**) Relative *PTGIS* mRNA expression in HUVECs treated subsequently with actinomycin D (ActD) for 8 h under normoxia and hypoxia (1% oxygen) (*n* = 5). *POLRIIa* mRNA expression was used as reference. Mean relative *PTGIS* mRNA expression in HUVECs treated subsequently with actinomycin D (ActD) for 8 h under normoxia and hypoxia (1% oxygen) (*n* = 5). *POLRIIa* mRNA expression was used as reference. Statistics: Normal (Gaussian) distribution was tested by Shapiro–Wilk normality tests. All data had Gaussian distributions. Data were then analyzed with *t*-tests or one-way ANOVAs followed by Tukey’s multiple comparisons tests or two-way ANOVA and Sidak’s multiple comparisons tests, respectively. * *p* < 0.05; ** *p* < 0.01; *** *p* < 0.001; **** *p* < 0.0001. Abbreviations: ActD, actinomycin D; B2m, beta-2 microglobulin; crtl, control; HUVECs, human umbilical vein endothelial cells; NOX4, NADPH oxidase 4; PTGIS, prostaglandin I2 synthase; POLRIIa, RNA polymerase II subunit A; shRNA, short hairpin RNA; TBP, TATA-box binding protein; untr, untreated; WT, wild-type.

We confirmed a significant increase in *NOX4* expression after 8 h of hypoxia in primary human umbilical vein endothelial cells (HUVECs), with sustained elevation at 16 h and 24 h (Figure 2A). This was accompanied by increased H_2_O_2_ release following hypoxia, which was abolished in cells with shNOX4 (Figure 2B). Additionally, aortas from wild-type (WT) mice revealed elevated *Nox4* mRNA expression after 24 h of hypoxia (Figure 2C). In HUVECs, the mRNA expression of prostaglandin I2 synthase (*PTGIS*) was significantly upregulated after 24 h of hypoxia (Figure 2D). Correspondingly, concentration of 6-keto-PGF1 alpha, the non-enzymatic hydration product of prostacyclin (PGI_2_), was increased in the supernatant of HUVECs after 24 h of hypoxia (Figure 2E). Aortas of WT mice indicated a significant upregulation of *Ptgis* mRNA expression after hypoxia, which was also highly significant in aortas under hypoxia from Nox4^−/−^ mice (Figure 2F). Experiments with Actinomycin D demonstrated similar degradation of *NOX4* mRNA under normoxic and hypoxic conditions (Figure 2G). Also, *PTGIS* mRNA degradation did not differ between normoxic and hypoxic conditions (Figure 2H), which contrasts *VEGF* mRNA stability, which is known to be increased under hypoxia (Supplementary Figure S1). Therefore, we obtained evidence that the regulation of the mRNA levels of *NOX4* and *PTGIS* in response to hypoxia are controlled at the level of gene transcription rather than by post-transcriptional processes like mRNA stability.

### 3.3. NOX4 and PTGIS Were Regulated by Different HIFs

**Figure 3:**
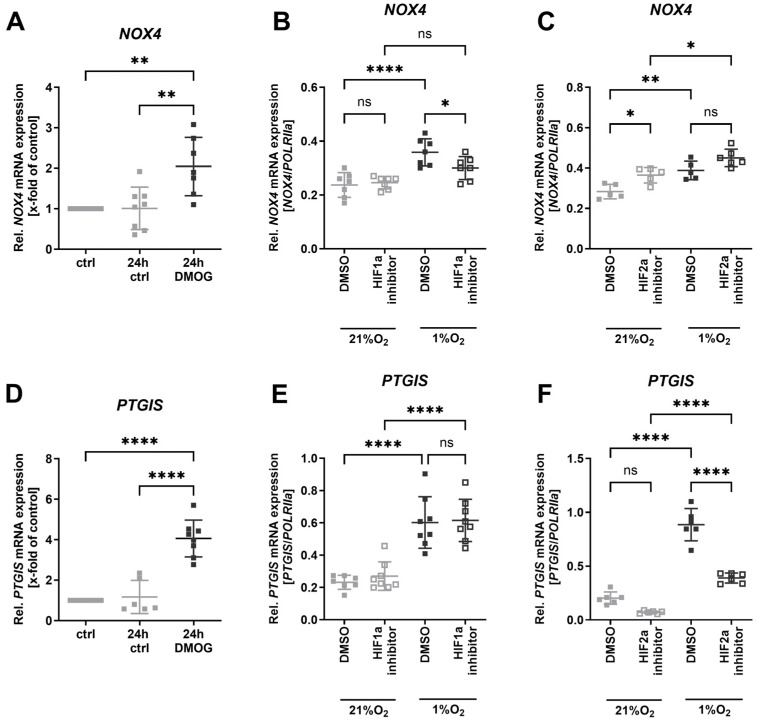
***NOX4* and *PTGIS* were predominantly regulated by different HIFs.** (**A**) Relative *NOX4* mRNA expression in HUVECs after 24 h 10 mM DMOG treatment under normoxic conditions (*n* ≥ 7). *TBP* mRNA expression was used as reference. (**B**) Relative *NOX4* mRNA expression in HUVECs treated subsequently with HIF1a inhibitor for 16 h under normoxia and hypoxia (1% oxygen) (*n* = 7). *POLRIIa* mRNA expression was used as reference. (**C**) Relative *NOX4* mRNA expression in HUVECs treated subsequently with HIF2a inhibitor for 16 h under normoxia and hypoxia (1% oxygen) (*n* = 5). *POLRIIa* mRNA expression was used as reference. (**D**) Relative *PTGIS* mRNA expression in HUVECs after 24 h 10 mM DMOG treatment under normoxic conditions (*n* ≥ 7). *TBP* mRNA expression was used as reference. (**E**) Relative *PTGIS* mRNA expression in HUVECs treated subsequently with HIF1a inhibitor for 16 h under normoxia and hypoxia (1% oxygen) (*n* = 8). *POLRIIa* mRNA expression was used as reference. (**F**) Relative *PTGIS* mRNA expression in HUVECs treated subsequently with HIF2a inhibitor for 16 h under normoxia and hypoxia (1% oxygen) (*n* = 6). *POLRIIa* mRNA expression was used as reference. Statistics: Normal (Gaussian) distribution was tested by Shapiro–Wilk normality tests. Non-Gaussian distributed data were analyzed by Kruskal–Wallis and Dunn’s multiple comparison tests (**D**,**E**). Gaussian distributed data (**A**,**B**,**C**,**F**) were analyzed with one-way ANOVA followed by Tukey’s multiple comparisons tests or two-way ANOVA and Sidak’s multiple comparisons tests, respectively. * *p* < 0.05; ** *p* < 0.01; **** *p* < 0.0001. Abbreviations: crtl, control; DMOG, dimethyloxallyl glycine; HIF, hypoxia-inducible factor; HUVECs, human umbilical vein endothelial cells; NOX4, NADPH oxidase 4; PTGIS, prostaglandin I2 synthase; POLRIIa, RNA polymerase II subunit A; TBP, TATA-box binding protein.

Treating human endothelial cells with prolyl-4-hydroxylase inhibitor dimethyloxalylglycine (DMOG) increases endogenous hypoxia-inducible factor (HIF) levels under normoxic conditions [26]. We detected elevated NOX4 (Figure 3A) and PTGIS (Figure 3D) expression in endothelial cells after DMOG application, indicating the HIF dependency of NOX4 and PTGIS upregulation under hypoxia. Inhibiting HIF1a under hypoxia resulted in decreased NOX4 expression (Figure 3B), but not PTGIS expression (Figure 3E). However, inhibiting HIF2a significantly prevented PTGIS upregulation under hypoxia (Figure 3F), while NOX4 was not regulated (Figure 3C).

### 3.4. Nox4 Released Hydrogen Peroxide Maintained Endothelial Function under Hypoxia

**Figure 4:**
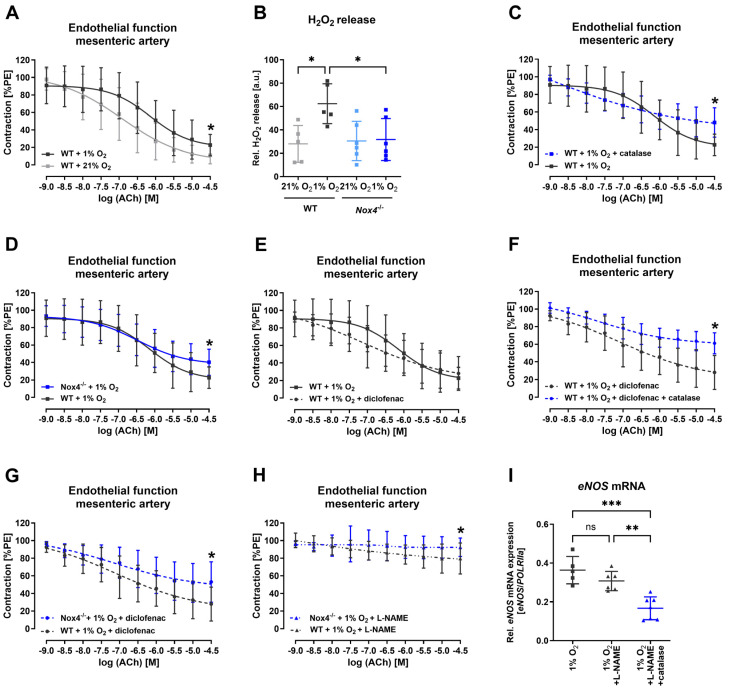
**H_2_O_2_ derived from Nox4 maintained vascular response of mesenteric arteries under hypoxia.** (**A**) Endothelium-dependent vasorelaxation of mesenteric arteries from WT mice under normoxic and hypoxic conditions ex vivo (*n* ≥ 11). (**B**) Hydrogen peroxide release in WT and Nox4^−/−^ mice mesenteric arteries ex vivo after hypoxia (*n* = 6). (**C**) Endothelium-dependent vasorelaxation of mesenteric arteries from WT mice and catalase treatment under hypoxic conditions ex vivo (*n* ≥ 6). (**D**) Endothelium-dependent vasorelaxation of mesenteric arteries from WT mice and Nox4^−/−^ mice under hypoxic conditions ex vivo (*n* ≥ 11). (**E**) Endothelium-dependent vasorelaxation of mesenteric arteries from WT mice and diclofenac treatment under hypoxic conditions ex vivo (*n* ≥ 6). (**F**) Endothelium-dependent vasorelaxation of mesenteric arteries from WT mice with diclofenac and catalase treatment under hypoxic conditions ex vivo (*n* ≥ 5). (**G**) Endothelium-dependent vasorelaxation of mesenteric arteries from WT mice and Nox4^−/−^ mice under hypoxic conditions and diclofenac treatment ex vivo (*n* ≥ 6). (**H**) Endothelium-dependent vasorelaxation of mesenteric arteries from WT mice and Nox4^−/−^ mice under hypoxic conditions and L-NAME treatment ex vivo (*n* ≥ 8). (**I**) Relative eNOS mRNA expression in HUVEC treated with hypoxia and L-NAME or L-NAME and catalase combination (*n* ≥ 5). *POLRIIa* mRNA expression was used as reference. Statistics: Normal (Gaussian) distribution was tested by Shapiro–Wilk normality tests. All data has Gaussian distribution. Data was then analyzed with *t*-tests or one-way ANOVAs, followed by Tukey’s multiple comparisons tests or two-way ANOVA and Sidak’s multiple comparisons tests, respectively. * *p* < 0.05; ** *p* < 0.01; *** *p* < 0.001. Abbreviations: Ach, acetylcholine; eNOS, endothelial nitric oxide synthase; L-NAME, N^G^-Nitro-L-arginine methyl ester; NOX4, NADPH oxidase 4; PE, phenylephrine; WT, wild-type.

To further assess the impact of hypoxia and Nox4 on vascular function, Mulvany myograph experiments were performed. Under hypoxic conditions in the organ chamber, vascular function of mesenteric arteries from WT and Nox4^−/−^ mice was analyzed. Endothelial vasorelaxation was declined in vessels under hypoxic conditions from WT mice (Figure 4A). We also detected an increased hydrogen peroxide release after hypoxia treatment in mesenteric arteries from WT mice, but not Nox4^−/−^ mice (Figure 4B). When we incubated the WT mesenteric arteries under hypoxic conditions with hydrogen peroxide scavenger catalase endothelial function was further altered (Figure 4C). Also, under hypoxic conditions in the organ bath chambers, endothelium-dependent vasorelaxation in the mesenteric artery of Nox4^−/−^ mice was further declined compared with WT mice (Figure 4D). In a next step, mesenteric arteries of WT mice under hypoxia were incubated with diclofenac to inhibit cyclooxygenases to gain insights on the impact of prostaglandins. Maximal vasorelaxation in WT mesenteric arteries was not affected by diclofenac under hypoxia (Figure 4E). However, mesenteric arteries of WT mice incubated with the combination of catalase and diclofenac showed an endothelial dysfunction (Figure 4F). Similarly, mesenteric arteries depleted of *Nox4* and incubated with diclofenac under hypoxia revealed an amplified endothelial dysfunction (Figure 4G). Incubating mesenteric arteries with nitric oxide synthase inhibitor L-NAME revealed that, also under hypoxic conditions, endothelial-dependent vasorelaxation was mainly mediated by nitric oxide, even though the impact was significantly increased in the mesenteric arteries of Nox4^−/−^ mice, indicating *Nox4* is relevant for endothelium-dependent vasorelaxation (Figure 4H). Additionally, incubating HUVECs with catalase and L-NAME showed the downregulation of *eNOS* mRNA expression (Figure 4I).

### 3.5. Restored Laminar Flow Prevented Upregulation of NOX4 and PTGIS under Hypoxia

**Figure 5:**
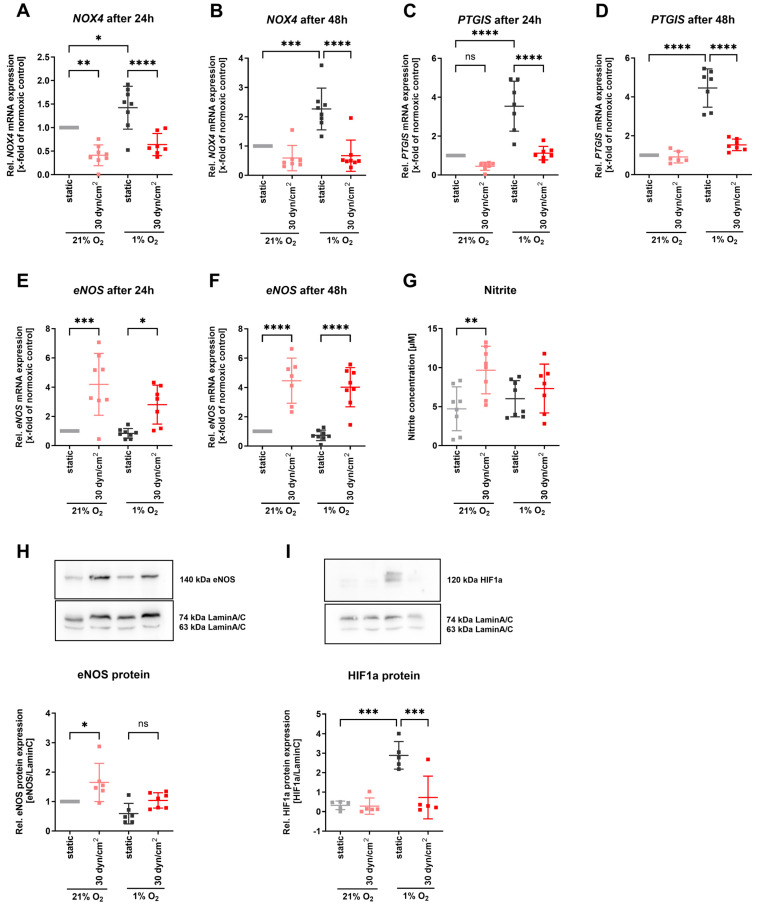
**Laminar flow of 30 dyn/cm^2^ on human endothelial cells prevented upregulation of NOX4 and PTGIS under hypoxia.** (**A**) Relative NOX4 mRNA expression in endothelial cells under laminar 30 dyn/cm^2^ flow after 24 h and (**B**) 48 h normoxic or hypoxic conditions (*n* = 8). TBP mRNA expression was used as reference. (**C**) Relative PTGIS mRNA expression in endothelial cells under laminar 30 dyn/cm^2^ flow after 24 h and (**D**) 48 h normoxic or hypoxic conditions (*n* = 8). TBP mRNA expression was used as reference. (**E**) Relative eNOS mRNA expression in endothelial cells under laminar 30 dyn/cm^2^ flow after 24 h and (**F**) 48 h normoxic or hypoxic conditions (*n* = 8). TBP mRNA expression was used as reference. (**G**) Nitrite concentration in supernatant of endothelial cells under laminar 30 dyn/cm^2^ flow after 24 h normoxic or hypoxic conditions (*n* = 8). (**H**) Relative eNOS protein expression of endothelial cells under static and laminar 30 dyn/cm^2^ flow after 24 h normoxic or hypoxic conditions (*n* = 6). (**I**) Relative HIF1a protein expression in endothelial cells under static and laminar 30 dyn/cm^2^ flow after 24 h hypoxic conditions (*n* = 5). Statistics: Normal (Gaussian) distribution was tested by Shapiro–Wilk normality tests. All data had Gaussian distributions. Data were then analyzed with two-way ANOVAs and Sidak’s multiple comparisons tests, respectively. * *p* < 0.05; ** *p* < 0.01; *** *p* < 0.001; **** *p* < 0.0001. Abbreviations: eNOS, endothelial nitric oxide synthase; HIF1a, hypoxia-inducible factor 1a; NOX4, NADPH oxidase 4; PTGIS, prostaglandin I2 synthase; TBP, TATA-box binding protein.

To study how protective blood flow affects the hypoxic upregulation of *NOX4* and *PTGIS*, we cultured human endothelial cells under hypoxia combined with 30 dyn/cm^2^ laminar flow to mimic restored blood flow. The elevated expression of *NOX4* under hypoxia was significantly diminished when treating endothelial cells additionally under laminar flow conditions for 24 and 48 h (Figure 5A,B). Similarly, *PTGIS* mRNA expression was significantly decreased under hypoxia when endothelial cells were cultured under protective laminar flow (Figure 5C,D). In contrast, laminar shear stress known to induce eNOS expression still led to upregulation of *eNOS* mRNA expression for 24 and 48 h when shear stress was applied under hypoxic conditions (Figure 5E,F). However, eNOS protein expression and nitric oxide release only tended to increase due to laminar flow under hypoxia conditions (Figure 5E,G). The other way around, we observed HIF1a protein expression being significantly diminished in those hypoxic endothelial cells where additional laminar flow was applied (Figure 5F).

## 4. Discussion

In the current study, we present evidence that NOX4 and PGI_2_ producing the PTGIS enzyme correlate under hypoxia. We observed NOX4 being regulated by HIF1a, while PTGIS was induced by HIF2a. We showed, for the first time, that NOX4 mediated endothelial function under hypoxia, and thereby complemented PGI_2_ in the vasculature under hypoxia. Restored laminar shear stress restored eNOS expression and blunted the hypoxic response on NOX4 and PTGIS.

We showed for the first time a tight correlation of NOX4 and PGI_2_ producing PTGIS enzyme expression in human occluded hypoxic vessels. In human endothelial cells, we observed an upregulation of NOX4 expression prior to the upregulation of PTGIS. The *NOX4* mRNA expression reached significant levels after 8 h. These data are in agreement with studies showing increased NOX4 expression in bovine and human endothelial cells exposed to hypoxia (1% oxygen) [27,28]. An increased NOX4 expression in response to hypoxia has also been shown in other cell types, including renal cells [6], neuronal cells [7], pulmonary arterial smooth muscle cells [8], pulmonary artery adventitial fibroblasts [9], embryonic stem cells [29], and adipose-derived stem cells [30]. In our experiments, NOX4 expression was only downregulated under hypoxia by the HIF1a inhibitor, while PTGIS expression was affected by HIF2a inhibition. Diebold et al. reported increased NOX4 expression in HIF1a-overexpressing cells [31]. However, other studies also indicated an impact of HIF2a on NOX4 [32]. It has also been reported that NOX4 might act upstream of HIF signaling. For example, in cardiomyocytes, it was shown that the NOX4-dependent upregulation of HIF1a caused increased angiogenesis via VEGF [19]. For PTGIS, in some studies, the regulation is attributed to HIF1a [33,34]; however, recent studies indicate both HIF1a and HIF2a are involved [32,35].

In the cardiovascular system, the role of NOX4 in hypoxia has always been tightly linked to angiogenesis rather than vascular function. Thus, our experimental settings focused on vascular function under hypoxia. We were able to detect a hypoxia-induced endothelial dysfunction in WT mesenteric arteries. However, in mesenteric arteries from *Nox4^−/−^* mice, the hypoxia-induced endothelial dysfunction was further altered. Inhibition of COX-1 and COX-2, leading to reduced PGI_2_, resulted in the most pronounced endothelial dysfunction in *Nox4^−/−^* mesenteric arteries under hypoxia. Therefore, our findings implicate an endothelium-dependent vasodilatory effect of Nox4 under hypoxic conditions, accommodating PGI_2_ [18].

The interplay of NOX4 and PGI_2_ under hypoxic conditions facilitates vascular adaptation to enhance tissue oxygenation and blood supply. When laminar protective blood flow is restored, the hypoxia-induced signaling is blunted. We noted that a laminar flow of 30 dyn/cm^2^ led to increased eNOS expression even under hypoxic conditions, resulting in decreased expression of HIFa and its target genes, *NOX4* and *PTGIS*. Others have shown that laminar shear stress also diminished hypoxia-induced apoptosis in HUVECs [36]. In human pulmonary endothelial cells, modification of hypoxia-induced signaling pathways due to laminar blood flow was also demonstrated [37]. Similarly, our results showed that hypoxia-induced NOX4 expression was inhibited by laminar shear stress. These observations might explain the controversial reports on NOX4 being involved in hypoxia-induced vascular diseases. Veith et al. could not detect any effect of *Nox4* deletion on pulmonary vasoconstriction and hypertension caused by hypoxia [38]. Other studies showed the upregulation of NOX4 in hypoxic pulmonary artery smooth muscle cells and animal models of hypoxia-induced pulmonary hypertension [39]. Shear stress–hypoxia interactions might account for these different observations.

Additionally, we found, that laminar shear stress-induced eNOS expression was accompanied by a reduced HIF1a expression. It has been reported that long-term exposure to nitric oxide, comparable to our experimental conditions, under flow has reducing effects on HIF1a accumulation and activity [40,41,42]. Mechanistically, it has been suggested that nitric oxide leads to the redistribution of oxygen towards PHDs by inhibiting cytochrome-c oxidase and thus mitochondrial respiration [43]. Cattaneo et al. also demonstrated that treatment of endothelial cells with nitric oxide synthase inhibitor N^G^-Nitro-L-arginine methyl ester (L-NAME) led to increased HIF1a stability and elevated HIF1a target gene expression [44]. Additionally, H_2_O_2_ from NOX4 can directly increase endothelial NO release via the AKT serine/threonine kinase 1-dependent phosphorylation of eNOS [45,46] or via the redox-sensitive Ca^2+^ channel transient receptor potential cation channel, subfamily M, member 2 (TRPM2) [47], again counteracting the hypoxic response.

## 5. Conclusions

For the first time, we demonstrated a tight correlation of NOX4 and PTGIS in endothelial cells under hypoxia in human occluded hypoxic vessels. NOX4 was essential to ensure endothelium-dependent vasorelaxation under hypoxia. Thus, NOX4, one of the main sources for hydrogen peroxide, might contribute to enhancing vascular function to restore blood circulation under hypoxia. Therefore, NOX4 inhibitors that are currently explored for the treatment of diseases such as fibrosis and certain cancers need to be verified for cardiovascular side effects.

## 6. Limitations

In this study, human femoral arteries, internal mammary arteries, as well as murine mesenteric vessels and aortas were investigated. The broad range of examined vessels also represents a limitation, as different factors have varying degrees of significance in the different types of vessels. Additionally, all human samples were obtained either from patients undergoing coronary artery bypass grafting surgery or those with peripheral vascular disease, limiting our ability to draw conclusions about human vessels under healthy physiological conditions.

The use of the Griess assay for nitrite measurement in our experiments presented certain methodological limitations. Specifically, nitrate was not accounted for, which may have led to an underestimation of total nitric oxide production.

Furthermore, our study focused on hydrogen peroxide released by NOX4. Other potential sources of hydrogen peroxide, such as mitochondrial dysfunction and eNOS monomerization, might play a role in our context and need to be addressed in further studies.

## Figures and Tables

**Table 1:** Patient data.

Parameter	
**Age**, years, median with range, *n*	73.0 (54–81), 11
**Sex**, male:female, % male	9:2, 81%
**BMI**, kg/m^2^, median with range, *n*	28.7 (22.9–39.9), 11
**Blood glucose**, mmol/L, median with range, *n*	5.12 (3.91–12.74), 8
**LDL cholesterol**, mmol/L, median with range, *n*	1.6 (1.17–3.26), 6
**HDL cholesterol**, mmol/L, median with range, *n*	1.04 (0.84–1.54), 5
**Total cholesterol**, mmol/L, median with range, *n*	3.24 (2.35–5.53), 6
**Triglycerides**, mmol/L, median with range, *n*	1.77 (1.36–2.98), 6
**CRP**, mg/L, median with range, *n*	7.40 (3.91–12.74), 8

**Table 2:** Primers used for real-time PCR.

Gene	Primers	Sequence, 5′-3′	
*B2m* murine	Forward	TCTCACTGACCGGCCTGTAT	NM_009735.3
	Reverse	GATTTCAATGTGAGGCGGGTG	
*eNOS* human	Forward	GAACCTGTGTGACCCTCACC	NM_000603.5; NM_001160109.2; NM_001160110.1; NM_001160111.1
	Reverse	TGGCTAGCTGGTAACTGTGC	
*NOX4* human	Forward	TAACCTCAACTGCAGCCTTATC	NM_001143836.3; NM_001143837.2; NM_001291926.2; NM_001291927.1; NM_001291929.2; NM_001300995.1; NM_016931.5
	Reverse	CTTTTATCCAACAATCTCCTGGTTCTC	
*Nox4* murine	Forward	TGTTGGGCCTAGGATTGTGTT	NM_001285833.1; NM_001285835.1; NM_015760.5
	Reverse	AGGGACCTTCTGTGATCCTCG	
*POLRIIa* human	Forward	ACCTGCGGTCCACGTTGTGT	NM_000937.4
	Reverse	CCACCATTTCCCCGGGATGCG	
*PTGIS* human	Forward	ACTGCCTGGGGAGGAGTTAT	NM_000961.4
	Reverse	GGGATCTCCACATCTGCGTT	
*Ptgis* murine	Forward	GTTGGTGGCGGTGACTTGTT	NM_001420752.1; NM_008968.5; NM_001420756.1;
	Reverse	CAGCATCTCTCCCAAACTCCA	
*TBP* human	Forward	CGCCGGCTGTTTAACTTCG	NM_003194.5
	Reverse	AGAGCATCTCCAGCACACTC	

**Table 3:** Antibodies used.

Primary Antibody	Company	Dilution	Molecular Weight
HIF1a	BD Biosciences	1:1000	120 kDa
eNOS	BD Biosciences	1:1000	140 kDa
Lamin A/C	Cell signaling	1:2000	74 kDa/63 kDa

## Data Availability

The raw data supporting the conclusions of this article will be made available by the authors on request.

## Supplementary Materials

The following supporting information can be downloaded. Supplementary Figure S1: VEGF mRNA expression as controls for experiments with DMOG treatment and actinomycin D; Supplementary Figure S2: No correlation of NOX4 and eNOS expression; Supplementary Figure S3: Endothelium-independent vasorelaxation.
